# Binding and Endocytosis of Bovine Hololactoferrin by the Parasite *Entamoeba histolytica*

**DOI:** 10.1155/2015/375836

**Published:** 2015-05-18

**Authors:** Guillermo Ortíz-Estrada, Víctor Calderón-Salinas, Mineko Shibayama-Salas, Nidia León-Sicairos, Mireya de la Garza

**Affiliations:** ^1^Departamento de Biología Celular, Centro de Investigación y de Estudios Avanzados del IPN (CINVESTAV-IPN), Avenida IPN 2508, 07360 México, DF, Mexico; ^2^Departamento de Bioquímica, CINVESTAV-IPN, Avenida IPN 2508, 07360 México, DF, Mexico; ^3^Departamento de Infectómica y Patogénesis Molecular, CINVESTAV-IPN, Avenida IPN 2508, 07360 México, DF, Mexico; ^4^Unidad de Investigación, Facultad de Medicina, Universidad Autónoma de Sinaloa, Cedros y Sauces, Fraccionamiento los Fresnos, 80246 Culiacán, SIN, Mexico

## Abstract

*Entamoeba histolytica* is a human parasite that requires iron (Fe) for its metabolic function and virulence. Bovine lactoferrin (B-Lf) and its peptides can be found in the digestive tract after dairy products are ingested. The aim of this study was to compare virulent trophozoites recently isolated from hamster liver abscesses with nonvirulent trophozoites maintained for more than 30 years in cultures *in vitro* regarding their interaction with iron-charged B-Lf (B-holo-Lf). We performed growth kinetics analyses of trophozoites in B-holo-Lf and throughout several consecutive transfers. The virulent parasites showed higher growth and tolerance to iron than nonvirulent parasites. Both amoeba variants specifically bound B-holo-Lf with a similar *K*_*d*_. However, averages of 9.45 × 10^5^ and 6.65 × 10^6^ binding sites/cell were found for B-holo-Lf in nonvirulent and virulent amoebae, respectively. Virulent amoebae bound more efficiently to human and bovine holo-Lf, human holo-transferrin, and human and bovine hemoglobin than nonvirulent amoebae. Virulent amoebae showed two types of B-holo-Lf binding proteins. Although both amoebae endocytosed this glycoprotein through clathrin-coated vesicles, the virulent amoebae also endocytosed B-holo-Lf through a cholesterol-dependent mechanism. Both amoeba variants secreted cysteine proteases cleaving B-holo-Lf. These data demonstrate that the B-Lf endocytosis is more efficient in virulent amoebae.

## 1. Introduction

*Entamoeba histolytica* is an extracellular parasitic protozoan that causes amoebiasis, an infection that affects humans worldwide. Cysts are the infective stage transmitted via the fecal-oral route through the intake of contaminated food and water. When cysts are ingested, they tolerate the acidic pH of the stomach, and excystation occurs in the terminal ileum, producing the invasive stage or trophozoites (amoebae).* E. histolytica* trophozoites adhere to and invade the mucosa of the large intestine, ultimately causing dysentery, ulcers, fever, and abdominal pain. In addition, by an unknown mechanism, trophozoites occasionally travel to the liver via the portal vein, producing amoebic liver abscesses (ALA), which can be fatal if not treated. Amoebae also invade other organs, especially the brain and lungs [1]. Amoebiasis is the third most common cause of death by parasites, particularly in developing countries [2, 3].

During evolution, pathogens developed diverse strategies to obtain iron from host iron-containing proteins and with very high host specificity in several cases [4]. In Gram-negative bacteria, members of the* Neisseriaceae* family and the* Moraxellaceae* family express surface receptors that are capable of specifically binding host iron-charged Lf (holo-Lf) and extracting the iron from this glycoprotein for growth. In mammalian cells, both iron-charged Lf (holo-Lf) and iron-lacking Lf (apo-Lf) can be taken up by the cell through the same receptor (LfR) on the cell surface. Research on both human Lf (H-Lf) and bovine Lf (B-Lf) has focused on the identification and characterization of the LfR in a variety of cell types. Specifically, the human lipoprotein receptor-related protein (HLRP) has been indicated to be a mitogenic receptor for B-Lf in osteoblastic cells [5]. Tanaka et al. [6] found that B-Lf and H-Lf bound to the same receptor in human Jurkat lymphoblastic T-cells. In fibroblasts, HLRP is required for B-Lf-enhanced collagen-gel contractile activity [7].

Parasites express diverse host iron uptake systems and pathogenicity mechanisms. In parasitic protozoa, iron acquisition systems have been poorly studied.* E. histolytica* trophozoites depend on host iron for their survival and expression of virulence. In axenic cultures, amoebae depend on iron from the medium for growth and use both Fe^3+^ and Fe^2+^ ions [8–10]. Interestingly, hamsters fed ferrous gluconate had high incidence and severity of liver lesions; therefore, iron is an important nutrient for this amoeba. In addition, patients suffering from ALA presented a hypoferremic state in the serum, confirming that nutritional immunity by iron is produced in amoebiasis [11]. Amoebae require approximately 100 *μ*M iron for growth; therefore, the parasite has developed mechanisms to scavenge iron from host iron-containing proteins. We established that human holo-Lf (H-holo-Lf) supported the growth of a nonvirulent variant of* E. histolytica* through several consecutive culture passages. H-holo-Lf was recognized by two proteins (45 and 90 kDa) located at the amoebic membrane. Subsequently, H-holo-Lf was endocytosed by a mechanism inhibited by filipin and trafficked via the endosomal/lysosomal pathway. In acidic lysosomes, iron from H-holo-Lf was most likely released and the protein degraded [12].

B-Lf is present practically without degradation in the large intestine and feces of babies fed with milk formula and in infants. The B-Lf molecule is found in its complete form in a low percentage in adults who drink dairy products [13, 14]. In this study, we show that* E. histolytica* trophozoites use B-holo-Lf for their growth* in vitro* and analyze the binding and endocytosis of this glycoprotein by the parasite. Two amoeba variants, a strain that has been maintained in axenic culture for more than 30 years and is unable to produce ALA in hamsters (nonvirulent amoebae) and trophozoites derived from this strain that have been continuously passed through ALA in hamsters (virulent amoebae), were compared.

## 2. Materials and Methods

### 2.1. Iron-Containing Proteins Used in This Work

B-apo-Lf was obtained from NutriScience (USA) containing 4.1% iron and was subsequently iron-saturated to obtain B-holo-Lf as previously reported [16]. The homogeneity of B-holo-Lf was confirmed with 10% SDS-polyacrylamide gels. Iron-charged human lactoferrin (H-holo-Lf) (95–100% of iron), human holo-transferrin (H-holo-Tf) (100% iron), and human and bovine hemoglobin (H-Hb and B-Hb) were obtained from Sigma, St. Louis, MO, USA.

### 2.2. Culture of Nonvirulent Amoebae

Trophozoites of the* E. histolytica* HM-1:IMSS strain were axenically grown in BI-S-33 medium (Dibico, Mexico) [15] supplemented with 16% (v/v) heat-inactivated bovine serum (BS) (Microlab, Mexico) and Tween 80-vitamin mix (*In vitro*, Mexico). The cultures were grown in glass screw-cap tubes at 37°C for 48 h. The tubes were placed on an ice bath for 15 min, and the amoebae were harvested by centrifugation at 500 g and washed twice in PBS, pH 7.4. Glass materials were treated with 2.0 M HCl for 24 h and rinsed six times with double-distilled water before their use. All chemicals were obtained from Sigma.

### 2.3. Culture and Maintenance of Virulent Amoebae

The induction of ALA in hamsters was performed in accordance with the International Norms of Care and Use of Laboratory Animals (NOM 062-ZOO-1999). The experiments were conducted at the Animal Care Unit of CINVESTAV-IPN, Mexico. In all cases, 2-month-old male Syrian Golden hamsters (*Mesocricetus auratus*) with an average weight of 100 g were used. To activate the virulence of* E. histolytica*, three hamsters were intraperitoneally anesthetized with sodium pentobarbital (Anestesal, Smith Kline, Mexico), and a hepatic lobe was inoculated with 1.5 × 10^6^ amoebae in 0.2 mL of BI-S-33 medium (from a culture of 48 h) [17]. The animals were euthanized at day 7. Subsequently, a liver fragment was excised under sterile conditions and transferred to a tube with culture medium supplemented with serum, streptomycin (500 *μ*g/mL), and penicillin (500 U/mL) and cultured for 24–48 h at 37°C. These virulent trophozoites were reinoculated into hamsters six times and then resuspended in culture medium without serum or antibiotics prior to use in the assays with B-holo-Lf.

### 2.4. Growth of* E. histolytica* Trophozoites in B-holo-Lf

The culture media and iron concentrations used are shown in Table 1. The low-iron medium was BI-S-33 without ammonium ferric citrate (AFC) and BS, with vitamins, treated with 5 g/mL of Chelex-100 resin to remove iron from the trace reagents. The resin was subsequently removed by filtration, and the medium was sterilized. This medium contained 6.5 *μ*M iron and is henceforth referred to as “low iron.” With the purpose of determining whether B-holo-Lf sustains the trophozoite growth, the amoebae were maintained in low iron for 4 h to diminish their iron reserves and to synchronize the culture [18]. Trophozoites (10^4^) were then inoculated into BI-S-33, into low iron plus serum (19 *μ*M Fe), or into this medium with different concentrations of B-holo-Lf added. All of the cultures were incubated at 37°C for 96 h. Cell viability was determined every 24 h by the exclusion of trypan-blue dye and observed under a light microscope in a Neubauer chamber. For successive cultures, amoebae (10^4^) were inoculated into BI-S-33, low iron, and low-iron containing BS and B-holo-Lf (100, 115, 125, and 135 *μ*M total iron) for 48 h. Consecutive transfers were conducted at least three times in the same medium.

### 2.5. Quantitative Determination of Iron

The BI-S-33 medium containing Fe^3+^ (from AFC) or low iron medium supplemented with B-holo-Lf was tested in order to determine the iron quantity. The iron was dissociated from the B-holo-Lf in acidic medium (50 mM citrate buffer, pH 2.2). Fe^3+^ released was reduced into Fe^+2^ by means of ascorbic acid (113.5 mM). Ferrous ions were complexed with tripyridyl-triazine (9.6 mM) in a blue colour compound and absorbance was measured at 595 nm. The intensity of the coloured complex formed was proportional to the iron concentration in the sample [19].

### 2.6. Interaction between B-holo-Lf and* E. histolytica* Trophozoites

To study the binding and endocytosis of B-holo-Lf in* E. histolytica*, the amoebae were maintained in low-iron medium for 4 h, incubated for different periods of time in the same medium containing FITC-B-holo-Lf (100 *μ*M Fe), and analyzed by flow cytometry and confocal laser-scanning microscopy. FITC-B-holo-Lf was prepared using fluorescein isothiocyanate (1.5 mg/mL; Sigma) in 50 mM sodium carbonate buffer (pH 9.5), which was added dropwise to the B-holo-Lf (10 mg/mL in 50 mM sodium bicarbonate, pH 8.3) with constant agitation for 2 h at room temperature (RT). To eliminate the unbound label, the conjugate was passed through a Sephadex G-25 column in PBS, pH 7.4.

*Flow Cytometry*. To first explore for the presence of a B-holo-Lf-binding protein in* E. histolytica* (*Eh*BholoLfbp) and test whether the binding/internalization depends on time and energy, virulent and nonvirulent amoebae (2 × 10^5^) in low-iron medium were incubated with FITC-B-holo-Lf for 1–5, 10, and 15 min at 6°C (only binding) or 37°C (internalization). Next, parasites were washed with PBS and fixed with 2% (w/v) (final concentration) paraformaldehyde (PFA). The amoebae were processed for fluorescence quantification using a flow cytometer (FACScan; Becton Dickinson, USA).

*Confocal Laser-Scanning Microscopy*. To localize the* Eh*BholoLfbp, we followed the preceding protocol; however, the amoebae were incubated with FITC-B-holo-Lf for 30 s and 30 min. The samples were similarly washed and fixed. Nonspecific binding was blocked with 1 M glycine for 15 min. The amoebae were mounted in Vectashield on glass slides and examined under a Leica confocal TCS-SP2 microscope (at least 20 optical sections were observed in three independent experiments, each performed in triplicate).

### 2.7. Internalization of Iron-Containing Proteins in* E. histolytica*

To determine whether virulent amoebae have a different capacity to internalize other iron-containing proteins in addition to B-holo-Lf more than nonvirulent amoebae, trophozoites (2 × 10^5^) were maintained in low-iron medium for 4 h, incubated for different periods of time at 37°C in the same medium containing 100 *μ*M Fe derived from four FITC-labeled proteins, H-holo-Lf, H-holo-Tf, B-Hb, or H-Hb, and analyzed by flow cytometry. To determine the specificity of the* E. histolytica* B-holo-Lf binding sites and further internalization, virulent and nonvirulent trophozoites were preincubated at 37°C for 30 min with 40-fold excess of the following five unlabeled proteins: B-holo-Lf, H-holo-Lf, H-holo-Tf, B-Hb, or H-Hb. Next, the amoebae were washed, fresh medium containing FITC-B-holo-Lf (100 *μ*M Fe) was added, and the mixture was incubated for 30 min. The samples were washed with PBS, fixed with PFA, and processed for fluorescence quantification by flow cytometry.

### 2.8. Effect of Ionic Strength and pH on B-holo-Lf Binding by* E. histolytica*

First, several cations concentrations and buffers with different pH values that would not affect cell viability were tested. The amoebae (10^6^) in low iron were preincubated at 6°C for 30 min with the following cations: 0–25 mM of CaCl_2_, FeCl_3_, MgCl_2_, and FeSO_4_ and with pH 2–8 buffers. The amoebae were washed with PBS, fresh medium containing FITC-B-holo-Lf (100 *μ*M total iron) was added, and the mixture was incubated for 30 min. The samples were washed, fixed with PFA, and analyzed by flow cytometry.

### 2.9. Determination of **K**_**d**_, **B**_max⁡_, **V**_max⁡_ and the Number of* Eh*BholoLfbp

To determine whether* E. histolytica* binds and internalizes B-holo-Lf, we used whole cells that were maintained in low-iron medium for 4 h. Next, the cells were incubated with several concentrations of FITC-B-holo-Lf (1–12,000 nM) at 6°C or 37°C for 30 min. Subsequently, the amoebae were washed with PBS and fixed with PFA. The samples were processed for fluorescence quantification by flow cytometry. Finally, to determine *K*_*d*_, *B*_max⁡_, and *V*_max⁡_ and estimate the average number of* Eh*BholoLfbp per cell, saturation kinetics were analyzed and Scatchard plots were generated.

### 2.10. Determination of the B-holo-Lf Internalization Pathway Using Inhibitors

First, the maximal concentration of inhibitor that would not affect cell viability was determined. The amoebae (2 × 10^5^) were preincubated at 37°C for 30 min in medium with the following inhibitors: 1–100 *μ*M NH_4_Cl, 0.5–10% (w/v) sucrose, 5–25 *μ*M chlorpromazine, 5–10 *μ*g/mL filipin, 25–100 *μ*g/mL nystatin, 7.5–20 mM methyl-*β*-cyclodextrin, 100–300 nM wortmannin, 5 *μ*M cytochalasin D, and 1-2 *μ*M colchicine. Next, the amoebae were incubated at 37°C for 30 min with fresh medium containing FITC-B-holo-Lf (100 *μ*M Fe). The samples were washed with PBS, fixed with PFA, and processed for fluorescence quantification by flow cytometry and confocal laser microscopy. At least 20 optical sections were observed in three independent experiments, each in triplicate. In all of the cases, the basal fluorescence was subtracted from the assayed values.

### 2.11. Determination of the Proteolytic Activity of* E. histolytica* Trophozoites against B-holo-Lf

To characterize the amoebic proteases that cleave B-holo-Lf, the previously reported method of López-Soto et al. [20] was used with several modifications. Both variants of amoebae (10^6^) were maintained in BI-S-33 or low-iron medium lacking BS and AFC for 4 h at 37°C. Next, the amoebae were harvested, centrifuged, and washed twice with PBS (pH 7.4). The cells from the pellet were collected separately from the supernatant (SN). The cells were disrupted by five cycles of freeze-thawing in PBS, and the content corresponded to the crude cell extract (CCE). The protein concentration was quantified by the method of Bradford [21]. The SN was precipitated with absolute ethanol (1 : 1 v/v), centrifuged, and finally passed through a 0.22-*μ*m Durapore membrane (Millipore, Bedford, MA). The CCE and SN proteins were maintained at 4°C and used immediately.

Protease activity was determined by electrophoresis of CCE and SN in 10% SDS-PAGE copolymerized with 0.1% (w/v) of B-holo-Lf as the substrate. The proteins from two variants of amoebae were loaded (40 *μ*g per well). Electrophoresis was performed at 100 V, for 2.5 h at 4°C. The gels were rinsed and incubated for 1 h with orbital agitation in 2.5% (v/v) Triton X-100. Next, the gels were incubated overnight at 37°C with one of the following buffer solutions containing 2 mM CaCl_2_ : 100 mM sodium acetate-Tris/HCl (pH 5.0), 100 mM Tris/NaOH (pH 7.0), or 100 mM glycine-Tris/NaOH (pH 9.0). Finally, the gels were stained with 0.5% Coomassie brilliant blue R-250 for 30 min. The protease activities were identified as clear bands on a blue background. To discern the type of proteolytic activity, CCE and SN were incubated for 1 h at 37°C with inhibitors at a final concentration of 10 mM* p*HMB, 5 mM NEM, or 10 *μ*M E-64 (cysteine-proteases); 2 mM EDTA (metallo-proteases); 5 mM PMSF (serine-proteases). The proteins were then separated by SDS-PAGE copolymerized with B-holo-Lf as mentioned above.

### 2.12. Statistical Analysis

All of the data are presented as the mean ± SD. The differences between the means in both groups of amoebae were compared using the *t*-test. The one-way analysis of variance (ANOVA) test was used to compare the difference between the means in more than two groups. A probability of *P* < 0.05 was taken to indicate statistical significance.

## 3. Results

### 3.1. Virulent Amoebae Are More Resistant to Stress Caused by Low and High Iron and Show Higher Growth in B-holo-Lf Than Nonvirulent Amoebae

**Figure 1:**
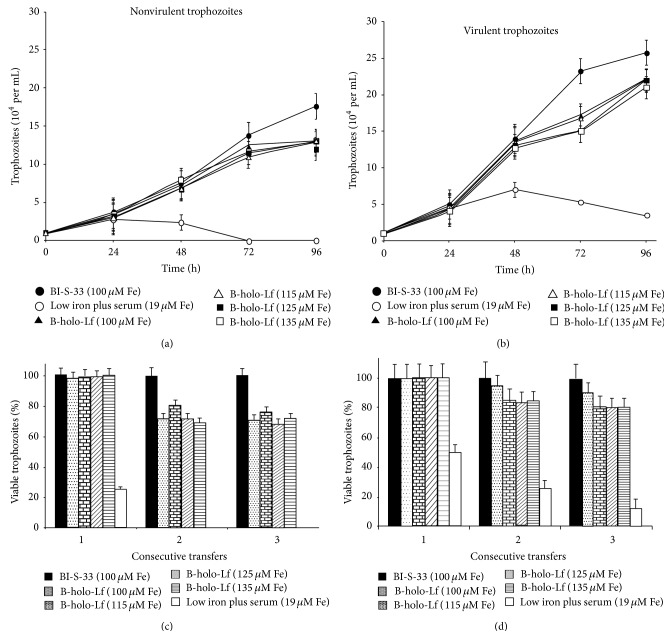
*E. histolytica* growth in B-holo-Lf as an iron source. ((a), (b)) Growth kinetics through 96 h in bovine holo-Lf (B-holo-Lf). Amoebic cultures were synchronized (see Methods section), 10^4^ cells were inoculated into each medium, and viability was estimated every 24 h by trypan blue exclusion. ((c), (d)) Amoebas were grown for 48 h, through three passages, in the media indicated. Viability was measured by trypan blue exclusion. Data are means of three independent experiments performed in triplicate ± SD.

To test the tolerance to stress caused by a low concentration of iron in* E. histolytica*, virulent and nonvirulent amoebae were grown in low-iron medium plus serum (19 *μ*M Fe). The nonvirulent amoeba culture developed slowly until 48 h, and no viable cells remained by 72 h (Figure 1(a)), indicating that iron is an essential element for the survival of this parasite and that this iron concentration is insufficient for growth, which are supported by previous reports from our group [12, 20]. In contrast, the virulent amoeba cultures showed a gradual reduction of the viable cell number until 96 h (Figure 1(b)); therefore, these amoebae were more resistant to the absence of iron. With respect to the tolerance to high iron concentration, we used up to 900 *μ*M Fe derived from AFC. Both amoeba variants tolerated this high iron concentration, but the virulent trophozoites used this metal in a more efficient manner (80–90% viability, data not shown) compared to the nonvirulent trophozoites (30% viability); this result supports previous results for nonvirulent amoebae [10].

To investigate whether B-holo-Lf supports the growth of* E. histolytica*, several concentrations of this glycoprotein were used as an iron source in growth kinetics analyses. B-holo-Lf (100–135 *μ*M Fe) supported the growth of both nonvirulent and virulent variants over a period of at least 96 h; however, the virulent amoebae were more efficient in using the iron from B-holo-Lf because an increase of approximately 70% in the number of amoeba was observed in this culture. However, the cultures of both amoeba variants showed higher growth in BI-S-33, in which the iron source is primarily ferric citrate, not in B-holo-Lf (Figures 1(a) and 1(b)). In addition, B-holo-Lf sustained subcultures of both variants of* E. histolytica*; however, the percentages of viable nonvirulent amoebae (Figure 1(c)) were lower throughout three consecutive culture passages than virulent amoebae (Figure 1(d)). With respect to the B-Lf iron used, virulent amoebae developed almost normally (90% viability in the third passage) at the four concentrations tested (Figure 1(d)). Furthermore, virulent amoebae were more tolerant of the stress caused by the absence of iron, as 10% of the cells were still viable at the third passage (Figure 1(d), white bar). Together, these results indicate that both variants are capable of growing in B-holo-Lf as an iron source, but virulent amoebae resist the variations in the iron concentration of the environment and apparently use B-holo-Lf more efficiently than nonvirulent amoebae.

### 3.2. *E. histolytica* Trophozoites Bind and Internalize B-holo-Lf through a Receptor

**Figure 2:**
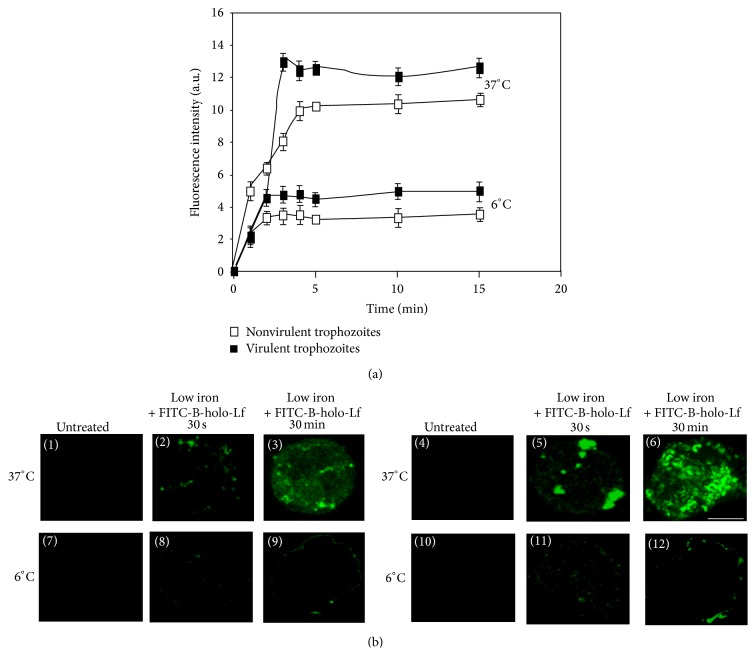
B-holo-Lf internalization in both variants of* E. histolytica* depends on time and energy. (a) Flow cytometry: amoebae were synchronized and incubated (2 × 10^5^ cells) at 37°C or 6°C with FITC-B-holo-Lf for the periods of time indicated. The samples were then fixed with PFA and processed for fluorescence quantification. Data are means of three independent experiments performed in triplicate ± SD. (b) Confocal microscopy: amoebae were synchronized and 2 × 10^5^ cells were incubated at 37°C or 6°C with FITC-B-holo-Lf for the periods of time indicated. 1–3 and 7–9 indicate the nonvirulent amoebae and 4–6 and 10–12 indicate the virulent amoebae. Bar, 10 *μ*m.

The binding and internalization of FITC-B-holo-Lf in* E. histolytica* were evaluated by incubation at 6°C and 37°C, respectively, using either flow cytometry (Figure 2(a)) or confocal microscopy (Figure 2(b)). In both amoeba variants, the binding and internalization were detected in the first minutes of incubation and reached saturation, suggesting the presence of B-holo-Lf-binding proteins (*Eh*BholoLfbp). The binding and saturation values were higher in the virulent amoebae (Figure 2(a), black squares). At 6°C and 30 minutes of incubation, we observed that FITC-B-holo-Lf bound to vesicle-like structures on the periphery (Figure 2(b), (panels 9 and 12)). FITC-B-holo-Lf is internalized at 30 minutes of incubation at 37°C (Figure 2(b), (panels 3 and 6)). Again, the virulent trophozoites showed higher binding and internalization of B-holo-Lf than the nonvirulent trophozoites. These results suggest that the B-holo-Lf internalization is time- and energy-dependent in* E. histolytica* and that an endocytosis mechanism could be involved in the uptake and utilization of B-holo-Lf by trophozoites. The B-holo-Lf internalization was continuous and saturable, and our results support the hypothesis that B-holo-Lf endocytosis is mediated by a protein receptor in* E. histolytica* and that this process may be regulated.

### 3.3. *E. histolytica* Trophozoites Specifically Endocytose B-holo-Lf

**Figure 3:**
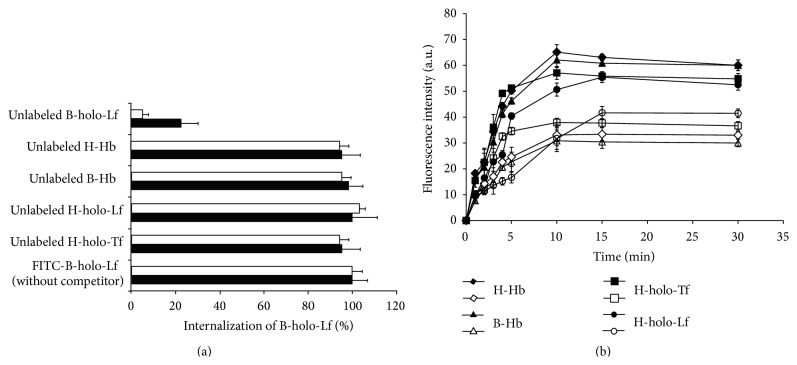
Both variants of* E. histolytica* endocytose B-holo-Lf specifically; however, virulent trophozoites show higher endocytosis level of iron-containing proteins than nonvirulent amoebae. (a) Amoebae (2 × 10^5^ cells) were synchronized and incubated at 37°C for 30 min with 40-fold excess of nonlabelled H-Hb, B-Hb, H-holo-Tf, H-holo-Lf, or B-holo-Lf. Later, the amoebae were washed and incubated with FITC-B-holo-Lf for 30 min and then processed for fluorescence quantification by flow cytometry. White bars indicate the nonvirulent amoebae and black bars indicate the virulent amoebae. Data are means of three independent experiments performed in triplicate ± SD. (b) Amoebae (2 × 10^5^ cells) were synchronized and incubated at 37°C with FITC-B-Hb, FITC-H-Hb, FITC-H-holo-Lf, and FITC-H-holo-Tf for the periods of time indicated. The samples were then fixed with PFA and processed for fluorescence quantification by flow cytometry. White symbols indicate the nonvirulent amoebae and black symbols indicate the virulent amoebae. Values are mean of three independent experiments performed in triplicate ± SD.

To determine whether the interaction of B-holo-Lf with both* E. histolytica* variants is specific for this glycoprotein, competition assays with other iron-containing proteins were developed. Virulent and nonvirulent amoebae were incubated with 40-fold excess of unlabeled B-holo-Lf, H-holo-Lf, H-holo-Tf, B-Hb, and H-Hb and then were incubated with FITC-B-holo-Lf at 37°C. Only unlabeled B-holo-Lf prevented the endocytosis of FITC-B-holo-Lf (Figure 3(a)), suggesting that nonvirulent and virulent* E. histolytica* trophozoites exhibit specific mechanisms to internalize B-holo-Lf, distinct from the other iron-proteins tested. Interestingly, through five experiments (and increasing to 50-fold excess of the B-holo-Lf concentration), nonvirulent amoebae showed almost 100% inhibition by unlabeled B-holo-Lf; however, only up to 80% inhibition of internalization was found for virulent amoebae, suggesting that B-holo-Lf may also be endocytosed by other nonspecific mechanisms in virulent amoebae.

### 3.4. Virulent* E. histolytica* Trophozoites Show Higher Endocytosis Capacity of Iron-Containing Proteins Than Nonvirulent Amoebae

To determine whether virulent trophozoites have higher capacity to internalize iron-containing proteins than nonvirulent trophozoites, internalization kinetics analyses at 37°C for four iron-proteins were performed by flow cytometry. The immediate internalization of FITC-B-Hb, FITC-H-Hb, FITC-H-holo-Tf, and FITC-H-holo-Lf was observed, achieving a maximum in the range of 5–10 min after the interaction with both variants of amoebae. Importantly, the internalization of these four iron-containing proteins was more rapid and efficient in the virulent amoebae than in nonvirulent amoebae in the following order: B-Hb and H-Hb (100% more at min 10), H-holo-Tf (50% more at min 10), and H-holo-Lf (30% more at min 15) (Figure 3(b)). In addition, in both variants, the internalization of all of these proteins was continuous and involved a prolonged saturation until 30 minutes of incubation. Together, these results suggest that virulent amoebae have a higher ability to bind and endocytose ferric- and ferrous-iron proteins to obtain iron, which may be an important virulence factor during infection.

### 3.5. *E. histolytica* Trophozoites Bind B-holo-Lf at Human Intestinal pH

**Figure 4:**
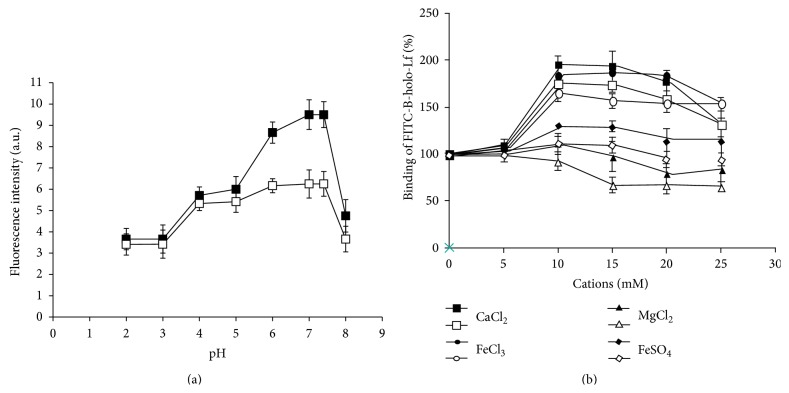
*Entamoeba histolytica* binding to B-holo-Lf is dependent of pH and it was increased in presence of Ca^2+^ and Fe^3+^. (a) Amoebae were synchronized and preincubated (10^6^ cells) with buffers (pH 2–8) at 6°C for 30 min. Next, fresh medium containing FITC-B-holo-Lf was added and incubated for 30 min. The samples were washed and fixed with PFA, and fluorescence quantification was analyzed by flow cytometry. (b) Amoebae (10^6^ cells) in low iron were preincubated at 6°C for 30 min with one of the following cations: 0–25 mM of CaCl_2_, FeCl_3_, MgCl_2_, or FeSO_4_. Next, amoebae were washed with PBS, and fresh medium containing FITC-B-holo-Lf was added and incubated for 30 min. The samples were washed and fixed with PFA, and fluorescence quantification was analyzed by flow cytometry. White symbols indicate the nonvirulent amoebae and black symbols indicate the virulent amoebae. Values are means of three independent experiments performed in triplicate ± SD.

To determine the optimum pH to reach the maximum ability of B-holo-Lf to bind in both variants of amoebae, the experiments were conducted at pH values between 2 and 8 at 6°C using whole cells and the binding was analyzed by flow cytometry. The optimum pH for B-holo-Lf binding ranged from 6.0 to 7.4 in both amoeba variants. In addition, B-holo-Lf binding drastically decreased in alkaline pH (pH = 8.0) (Figure 4(a)). This finding suggests that B-holo-Lf binding (and most likely endocytosis) by amoebae occurs at the pH of the human intestine, which should play an important role in the binding and use of B-holo-Lf by amoebae. These results also suggest a defined and optimum pH at which the maximum amount of B-holo-Lf binds to the amoebae, which supports the notion of specific binding proteins for this glycoprotein.

### 3.6. Calcium and Ferric Iron Increase the Binding of B-holo-Lf in* E. histolytica*

To determine the effect of the ionic strength and ion specificity on B-holo-Lf binding in both variants of amoebae, binding assays (6°C) in the presence of cations were developed, and the fluorescence was quantified by flow cytometry. In total, a 100% increase in B-holo-Lf binding in the presence of Ca^2+^ and Fe^3+^ was observed in both amoeba variants compared to the untreated amoebae. Fe^2+^ and Mg^2+^ did not have an effect on B-holo-Lf binding (Figure 4(b)). The binding of this glycoprotein was not affected when the amoebae were incubated with the monovalent cations Na^+^ and K^+^ (data not shown).

### 3.7. Virulent* E. histolytica* Trophozoites Show Higher **B**_max⁡_, **V**_max⁡_, and Number of Binding Sites for B-holo-Lf Than Nonvirulent Amoebae

**Figure 5:**
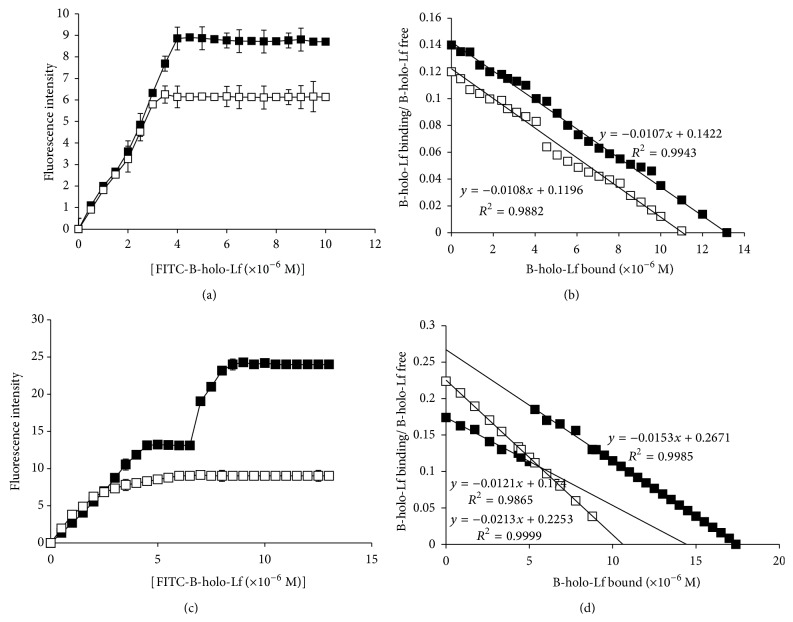
*E. histolytica* trophozoites bind and internalize B-holo-Lf with high affinity; however, virulent amoebae show higher *V*_max⁡_ and number of* Eh*BholoLfbp than nonvirulent amoebae. ((a), (c)) Amoebae (5 × 10^5^ cells) were synchronized and incubated with several concentrations of FITC-B-holo-Lf (1–12,000 nM) at 6 or 37°C for 30 min. The samples were washed and fixed with PFA, and fluorescence quantification was analyzed by flow cytometry. Data are means of three independent experiments performed in triplicate ± SD. ((b), (d)) Determination of *K*_*d*_, *B*_max⁡_, *V*_max⁡_, and the number of* Eh*BholoLfbp/cell was estimated by the method of Scatchard. In all cases, white and black squares indicate the nonvirulent amoebae and virulent amoebae, respectively. Data are means of three independent experiments performed in triplicate ± SD.

The maximum binding capability of a ligand depends on the affinity of the cell binding proteins for the ligand, which is understood by saturation kinetics. To determine whether* E. histolytica* binds B-holo-Lf with constant kinetics and in a saturable manner, we used whole cells that were incubated with several concentrations of FITC-B-holo-Lf at 6°C and analyzed the binding by flow cytometry. As shown in Figure 5(a), the* E. histolytica* binding kinetics to B-holo-Lf were saturable with one kinetic component involved in each amoeba variant. The binding was similar and increased up to 3.25 *μ*M for nonvirulent amoebae and up to 3.96 *μ*M for virulent amoebae. Furthermore, during the internalization at 37°C using the same concentrations of B-holo-Lf, one kinetic component that participates in the accumulation of B-holo-Lf in nonvirulent amoebae was found; however, two kinetic components were involved in the internalization of this glycoprotein in virulent amoebae (Figure 5(c)). These results suggest the presence of a specific saturable mechanism that allows the binding/internalization of B-holo-Lf and supports the results previously mentioned. Additionally, the virulent amoebae showed a more efficient system for the binding and internalization of B-holo-Lf than nonvirulent amoebae.

Subsequently, Scatchard plot transformation for FITC-B-holo-Lf was obtained from analyses of nonlinear regression, which is shown in Figures 5(b) and 5(d) and Table 2. Both amoebae specifically bound B-holo-Lf with a similar apparent *K*_*d*_ (1.85 × 10^−6 ^M and 2.3 × 10^−6 ^M for nonvirulent and virulent amoebae, resp.). However, the maximal binding at 6°C (*B*_max⁡_) for B-holo-Lf was higher in virulent amoebae (1.659 *μ*mol/min) compared to nonvirulent amoebae (0.066 *μ*mol/min) (Figure 5(b)). In addition, the maximal velocity of internalization at 37°C (*V*_max⁡_) was higher and showed two kinetic components in virulent amoebae (3.3 and 0.9 *μ*mol/min) than in nonvirulent amoebae (0.4562 *μ*mol/min), which only showed one component (Figure 5(d)). These data indicate that the binding affinity of B-holo-Lf was similar to other pathogenic protozoa; however, virulent amoebae showed higher *B*_max⁡_ and *V*_max⁡_ than nonvirulent amoebae, suggesting that virulent amoebae are capable of binding higher concentrations of B-holo-Lf, and this mechanism was more efficient for the binding of this glycoprotein than nonvirulent amoebae. Importantly, an average of 9.45 × 10^5^ (nonvirulent amoebae) and 6.65 × 10^6^ (virulent amoebae) binding sites for B-holo-Lf per amoeba were found (Table 2).

### 3.8. B-holo-Lf Is Primarily Endocytosed via Clathrin-Coated Vesicles by* E. histolytica* Trophozoites

**Figure 6:**
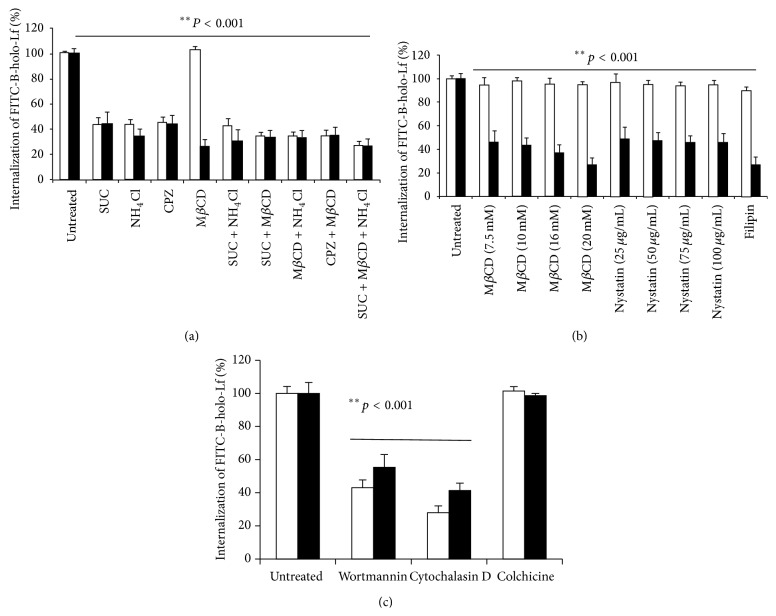
B-holo-Lf is primarily endocytosed via clathrin-coated vesicles in both variants of* E. histolytica*; however, virulent trophozoites also endocytose B-holo-Lf through a cholesterol-dependent route. ((a) and (c)) Amoebae (2 × 10^5^ cells) in low iron were preincubated at 37°C for 30 min in medium with one of the indicated inhibitors. Next, they were incubated for 30 min with fresh medium containing FITC-B-holo-Lf. The samples were fixed and processed for fluorescence quantification by flow cytometry. (b) Amoebae were preincubated for 30 min at the concentration indicated of lipid-rafts inhibitors and processed as in (a). In all cases, white and black bars indicate the nonvirulent amoebae and virulent amoebae, respectively. Values are means of three independent experiments performed in triplicate ± SD.

To determine the cellular mechanism through which B-holo-Lf is taken up and internalized by both variants of amoebae, we used several types of inhibitors of endocytosis pathways, and B-holo-Lf internalization was evaluated by flow cytometry (Figure 6(a)). If the FITC-B-holo-Lf endocytosis in untreated amoebae is taken as 100%, the three clathrin-mediated endocytosis inhibitors, hypertonic sucrose solution, NH_4_Cl, and the cationic amphiphilic drug chlorpromazine, alone or in combination, inhibited the internalization of B-holo-Lf by 60% in virulent as well as nonvirulent amoebae (Figure 6(a)). Furthermore, inhibitors of cholesterol-mediated endocytosis, such as methyl-*β*-cyclodextrin (M*β*CD), filipin, and nystatin, did not inhibit B-holo-Lf endocytosis in nonvirulent amoebae (<5%) (Figure 6(b), white bars). However, B-holo-Lf endocytosis was affected by these three inhibitors in all concentrations tested (60–70%) in the* E. histolytica* virulent variant (Figure 6(b), black bars). These results suggest that B-holo-Lf endocytosis occurs through both clathrin-coated-vesicles and a cholesterol-dependent mechanism in virulent amoebae. Wortmannin and cytochalasin D considerably affected B-holo-Lf endocytosis in both variants (43 and 40% inhibition, resp.) (Figure 6(c)). However, colchicine had a minor effect (15% inhibition). These results suggest that B-holo-Lf endocytosis may depend on PI3K activity and the actin cytoskeleton and not on microtubules.

### 3.9. *E. histolytica* Trophozoites Degrade B-holo-Lf through Cysteine Proteases

**Figure 7:**
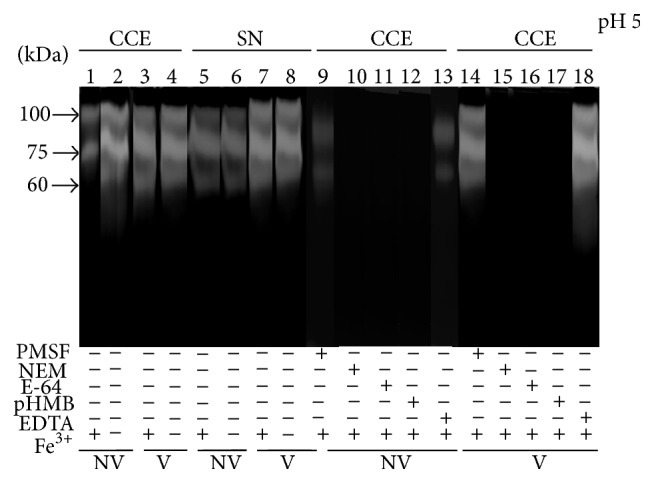
*E. histolytica *trophozoites degrade B-holo-Lf by means of cysteine proteases. Amoebae (10^6^ cells) were maintained in BI-S-33 or low-iron medium for 4 h at 37°C. Next, the amoebae were harvested and washed and cells from the pellet were disrupted by freeze-thawing. Proteins from crude cell extract (CCE) and culture supernatant (SN) were separated by electrophoresis on a 10% SDS-PAGE copolymerized with 0.1% of B-holo-Lf. After that, the gels were incubated with a buffer (pH 5.0) and stained with Coomassie blue. CCEs of nonvirulent amoebae (NV) and virulent amoebae (V) were treated with the protease inhibitors indicated (lanes 9–18). Result is representative of three independent experiments.

Proteins that were secreted into the culture supernatant (SN) and were present within the cells (CCE) were analyzed by electrophoresis in substrate gels in both variants of amoebae. B-holo-Lf was cleaved at a wide range of pH values by three proteases with MWs of 100, 75, and 60 kDa from the CCE and SN in both variants of amoebae maintained with Fe^3+^-citrate (BI-S-33) and low-iron medium. The proteolytic pattern appears to be similar in CCE and SN in both variants of amoebae; therefore, they are most likely the same enzymes (activity at pH 5 is shown in Figure 7). Interestingly, our results show proteolytic activity against B-holo-Lf in the SN of* E. histolytica* cultures, unlike H-holo-Lf, where no proteolytic activity was detected in the SN of nonvirulent trophozoites [12]. Using B-holo-Lf as an in-gel substrate, the absence of iron increased the proteolytic activity of the three proteases in both variants of amoebae. Importantly, we found higher proteolytic activity (2-fold) in the CCE and SN of virulent amoebae compared with nonvirulent amoebae. Proteases against B-holo-Lf were cysteine-related, as NEM, E-64, and* p*HMB inhibited the cleavage activity of CCE and SN in both variants of amoebae (Figure 7). These data suggest that one of the mechanisms of* E. histolytica* to acquire iron from B-holo-Lf may be through internal and extracellular cysteine proteases.

## 4. Discussion

Iron (Fe) is an essential nutrient for both the host and the pathogens surviving inside the host. However, iron is toxic and leads to the production of free radicals via Fenton's reaction; thus, iron is generally bound to or forms part of proteins, and a less significant labile, low-molecular-mass pool of intracellular iron exists [22]. Due to the toxicity and as a general strategy against pathogens, mammals have evolved complex iron-withholding systems to prevent microbial growth [23]. Therefore, pathogens that seek to colonize the host encounter an iron-limiting environment [24–26], and the free-iron concentration in fluids is approximately 10^−18 ^M, an amount far too low to support their growth, which requires levels in the micromolar range [24]. Furthermore, several protozoa, such as amitochondriate protists (i.e.,* Trichomonas, Tritrichomonas, Giardia,* and* Entamoeba*), require particularly high amounts of iron for* in vitro* growth (50–200 *μ*M), surpassing the concentration in the majority of eukaryotic and prokaryotic cells (0.4–4 *μ*M). As a result, competition for iron between the host and pathogen occurs during infections [27, 28].

According to our results, amoebae recently obtained from ALA (virulent) tolerated higher iron concentrations and were able to grow through several consecutive transfers in the absence of iron compared to nonvirulent amoebae, which strongly suggests that a highly efficient iron storage mechanism may be involved in their survival. In pathogenic protozoa, a ferritin-like molecule has not been described. In* E. histolytica,* the presence of a labile low molecular-mass iron pool or specific compartments for iron storage, such as those described in mammalian cells, has not been reported [22, 29]. Virulent amoebae were recently extracted from liver abscesses and apparently the iron utilization may be more efficient than in nonvirulent counterparts.

Bovine lactoferrin is present practically without degradation in the feces of babies fed with milk formula and in infants; therefore, the B-Lf molecule can resist the acidic pH of the stomach [13]. Based on our results* in vitro*, B-holo-Lf provides the required iron for the growth of* E. histolytica*, and the amoebae were able to bind and internalize this glycoprotein. Furthermore, trophozoites recently isolated from liver abscesses showed high efficiency in the binding and endocytosis of B-holo-Lf, which suggests that these amoebae possess more binding sites or a higher affinity for this iron source than the nonvirulent parasites. Virulent variant obtained from hamster ALA should be similar to the amoebae causing human infection. These results allow us to hypothesize that virulent trophozoites may efficiently use B-holo-Lf* in vivo*. We have measured the concentration of Lf by ELISA in samples of commercial pasteurized bovine products: milk, yogurts, and powder milk for infants [30]. The Lf concentration fluctuates, depending on the mark: 10–90, 20–70, and 2–14 *μ*g/mL, respectively. In addition, in many countries, products such as cereals, milk, and cocoa are fortified with iron. Lönnerdal et al. [31] found 0.2-0.3 *μ*g/mL of iron in bovine milk, and this value increased to 4–12 *μ*g/mL in iron-fortified formula [32]. The normal iron saturation in Lf of milk is 30% and Lf could be more saturated in iron-fortified milk. In addition to lactoferrin, lactoperoxidase, casein, catalase and fat, bind iron. Iron from the diet and B-Lf could be increasing the possibility of infection.

The use of several iron-containing proteins such as H-holo-Tf, H-holo-Lf, H-Hb, HS-Ft, and Heme as sole sources of iron for the strain HM1-1:IMSS, which has been cultured* in vitro* for more than 30 years [12, 20, 33–36] has been described. In this work, we found that the binding of B-holo-Lf, H-holo-Lf, B-Hb, H-Hb, and H-holo-Tf increased in amoebae that recently passed through hamster livers. Several pathogenic protozoa are capable of recognizing iron-containing proteins through multifunctional proteins, including* Toxoplasma gondii* tachyzoites, which bind B-holo-Lf, B-holo-Tf, and chicken holo-ovo-Tf through one 42-kDa protein, suggesting low-specificity binding [37]. Regarding our results, we found that both variants of amoebae recognize B-holo-Lf with high specificity, and H-holo-Lf and H-holo-Tf did not compete with B-holo-Lf for the* Eh*BholoLf-binding sites despite the high homology in the amino acid sequence, which are approximately 77% between B-Lf and H-Lf and 60% between B-Lf and H-Tf [38, 39].

The maximum binding of B-holo-Lf by Lfbps or receptors in mammalian cells is dependent on environmental conditions such as pH, ionic strength, and temperature. In this work, the optimum pH for B-holo-Lf binding ranged from 6.0 to 7.4 in both amoeba variants. These results suggest that trophozoites may use B-holo-Lf in the human ileum and large intestine (pH = 7.2–7.6). In addition, the binding of Lf requires the presence of a calcium ion in some biological systems, especially in human enterocytes and rat hepatocytes, which bind H-Lf and B-Lf through Ca^2+^-dependent intelectin. Importantly, we found that the binding of B-holo-Lf increased in the presence of Ca^2+^ and Fe^3+^ in both variants of amoebae. Perhaps the presence of Ca^2+^ is necessary to achieve maximal binding of this glycoprotein [40].

Holo-Lf-binding proteins (holoLfbps) have been reported in several protozoan species, and each parasite possesses one or several characteristic proteins for capturing iron from this glycoprotein. In this work, relevant information regarding the affinity of the amoebae to bind and endocytose B-holo-Lf is reported. We found that both variants of* E. histolytica* bind and internalize B-holo-Lf with high affinity although on the micromolar order. The *K*_*d*_ was similar to that of H-holo-Lf (0.47–5.27 *μ*M) in* T. vaginalis* and to that of B-holo-Lf (3.6 *μ*M) in* T. foetus* [41–43]. In addition, B-holo-Lf efficiently accumulated in both amoeba variants at 37°C, but the virulent variant showed higher endocytosis than the nonvirulent amoebae, which suggests that virulent amoebae are more capable of using the iron from B-holo-Lf than their nonvirulent counterparts and that expression of at least two classes of specific binding sites for B-holo-Lf may occur. Importantly, virulent amoebae showed a higher *B*_max⁡_ (25-fold) and* Eh*BholoLfbps/per cell (7-fold) than nonvirulent amoebae. The number of sites/cell was comparable to the number determined in mammalian cells but higher as compared to other parasitic protozoa such as* T. vaginalis* and* T. foetus* [42, 43].

Higher eukaryotic cells take up nutrients by endocytic mechanisms, and clathrin is a protein that participates in the endocytic pathway of many molecules. Clathrin has recently been observed in protozoa such as* T. brucei* [44, 45],* T. cruzi* [46],* Leishmania major* [47],* Giardia lamblia* [48, 49], and* E. histolytica* [20, 34]. In this work, we found that clathrin-coated pit inhibitors blocked the B-holo-Lf internalization in both variants of amoebae. In addition, inhibitors of cholesterol-mediated endocytosis inhibited the B-holo-Lf endocytosis in the virulent variant of* E. histolytica*, suggesting that B-holo-Lf endocytosis is dependent on both endocytosis pathways only in virulent amoebae. In such amoebae, B-holo-Lf could also be internalized by using dynamin-independent or fluid-phase pathways, although to a lesser extent. Also, wortmannin, an inhibitor of the activity of PI3K, inhibited the B-holo-Lf endocytosis. In* E. histolytica*, PI3K is involved in vital events such as phagocytosis and pinocytosis [50–53]. Furthermore, in this work, we found that B-holo-Lf endocytosis decreased in both variants of amoebae treated with cytochalasin D, an inhibitor of actin polymerization. During endocytosis, actin microfilaments are crucial for the formation and movement of vesicles. The connection between receptor-mediated endocytosis and the actin cytoskeleton during the formation and detachment of newly formed vesicles is well documented in other cells [54–56]. In* E. histolytica*, actin has been linked to phagocytosis [52], fluid-phase endocytosis [57], exocytosis [58], and erythrophagocytosis [53]. In contrast, microtubules appear not to be involved in B-holo-Lf endocytosis by amoebae. However, microtubules are involved in the endocytosis of holoLf in mammalian cells where clathrin-dependent vesicles are organized by microtubules [59]. In* E. histolytica*, microtubules have been studied to determine their involvement in cell division, but the data have not been able to correlate microtubules with the movement of vesicles [60]. Several investigators have reported the effect of cholesterol in the virulence of amoebae. Serrano-Luna et al. cultured nonvirulent and virulent variants of the strain HM1:IMSS in the presence of cholesterol and observed that the nonvirulent amoebae slightly increased the endocytosis of latex microspheres [61]. Virulent amoebae, which continuously are in contact with cholesterol, showed similar endocytosis. In addition, HSF cells stimulated with cholesterol increased the caveolae-dependent endocytosis [62]. Although the gene of caveolin has not been found in* E. histolytica*, H-holo-Lf is cholesterol-dependent endocytosed in nonvirulent amoeba [12] and according to our results, B-holo-Lf is endocytosed through clathrin- and cholesterol-dependent vias in virulent amoeba. More experiments are needed to understand the effect of cholesterol in the endocytosis of iron-containing proteins by* E. histolytica*.

Proteases secreted by pathogenic protozoa are essential for their biology, including development, immune evasion, and host tissue degradation for nutrient acquisition. Iron acquisition from B-holo-Lf and H-holo-Lf, respectively, has been reported in two protozoan species:* T. foetus* [63] and* E. histolytica* [12]. In this work, both* E. histolytica* variants degraded B-holo-Lf through three internal cysteine proteases, and proteolytic cleavage of holoLf caused the release of iron for cellular metabolism. These results allow us to hypothesize that after being endocytosed, B-holo-Lf proteolysis may occur in amoebic lysosomes (pH < 4) where B-holo-Lf is degraded by proteases and iron is released in these organelles as previously suggested for iron acquisition from H-holo-Lf in nonvirulent amoebae [12]. The acidic environment of amoebic vesicles and the presence of cysteine proteases may be factors that contribute to B-Lf-iron release to support amoeba growth* in vitro*; however, this mechanism may also likely occur with B-holo-Lf during intestinal amoebiasis and ALA. Interestingly,* E. histolytica* trophozoites also degraded B-holo-Lf through secreted proteases to the culture supernatant, suggesting that this substratum may be cleaved before endocytosis, providing iron for parasite growth.

## 5. Conclusion

In this study, we compare the utilization of B-holo-Lf as an iron source by* E. histolytica* trophozoites of different virulence, for their growth* in vitro.* The results suggest that B-holo-Lf is specifically and rapidly captured by amoebic Lfbps with a very high affinity, and virulent amoebae showed higher *V*_max⁡_ and* Eh*BholoLfbps than nonvirulent amoebae. B-holo-Lf was internalized through constitutive and organized endocytic processes involving clathrin-coated pits and cholesterol. These data allow us to hypothesize that the endocytosis of iron-containing proteins from the human host as well as from bovine milk products may be important for the parasite.

## Figures and Tables

**Table 1:** Media used in this study.

Medium	Iron concentration (*μ*M)^∗^	Source of iron	Reference
BI-S-33	100 ± 4.68	AFC, serum, and iron traces from reagents	Diamond et al., 1978 [15]
Low-iron	6.5 ± 2.61	Iron traces from reagents	Serrano-Luna et al., 1998 [10]
Low-iron plus serum	19 ± 4.35	Serum and iron traces from reagents	Serrano-Luna et al., 1998 [10]
Low-iron plus serum and B-holo-Lf^▪^	100 ± 2.5, 115 ± 1.5, 125 ± 1.95 and 135 ± 1.85	Bovine Lf-iron, serum, and iron traces from reagents	[This work]

^*^Iron concentration was measured by spectrophotometric method. Micro-Tec Laboratory, Mexico.

^▪^B-holo-Lf was added to obtain the iron concentrations indicated.

**Table 2:** Biochemical properties of binding/internalization of B-holo-Lf in *E. histolytica*.

	6°C			37°C		
	*K*_*d*_	*B*_max⁡_	*Eh*BholoLfbp number	*K*_*d*_	*V*_max⁡_	*Eh*BholoLfbp number
	(M ± SD)^a^	(*μ*mol·min^−1^± SD)^a^	(sites/cell ± SD)^a^	(M ± SD)^a^	(*μ*mol·min^−1^± SD)^a^	(sites/cell ± SD)^a^
Nonvirulent amoebae	1.85 ± 0.002 × 10^−6^	0.066 ± 0.0001	9.45 ± 0.093 × 10^5^	2.5 ± 0.002 × 10^−6^	0.4562 ± 0.00012	1.89 ± 0.00023 × 10^7^
Virulent amoebae	2.3 ± 0.0015 × 10^−6^	1.659 ± 0.0013	6. 65 ± 0.011 × 10^6^	2.48 ± 0.0012 × 10^−6^	3.3 ± 0.0016	1.97 ± 0.00043 × 10^7^
				5.2 ± 0.0027 × 10^−6^	0.9 ± 0.00045	

^a^Three independent experiments were done by triplicate.

SD: standard deviation.
